# Correlation of BMI With Potential Barriers to Mammographic Screening: Body Image, Pain, and Perceived Risk of Breast Cancer–A Cross‐Sectional Study

**DOI:** 10.1111/1754-9485.70085

**Published:** 2026-03-19

**Authors:** Elizabeth Dzidzornu, Nina S. McCarthy, Kate McBride, Elizabeth Stewart, Julie Wilkes, Ann White, Martha Hickey, Emmeline Lee, Jennifer Stone

**Affiliations:** ^1^ School of Population and Global Health University of Western Australia Perth Western Australia Australia; ^2^ School of Biomedical Sciences, University of Western Australia Perth Western Australia Australia; ^3^ Translational Health Research Institute, Western Sydney University Penrith New South Wales Australia; ^4^ School of Medicine, Western Sydney University Penrith New South Wales Australia; ^5^ BreastScreen Victoria Melbourne Victoria Australia; ^6^ BreastScreen Western Australia Perth Western Australia Australia; ^7^ Women and Newborn Health Service King Edward Hospital Perth Western Australia Australia; ^8^ Australian Breast Density Consumer Advisory Council Perth Western Australia Australia; ^9^ Department of Obstetrics, Gynaecology and Newborn Health The University of Melbourne, VIC, and the Royal Women's Hospital Melbourne Victoria Australia; ^10^ Medical School, University of Western Australia Perth Western Australia Australia; ^11^ Sir Charles Gairdner Hospital Perth Western Australia Australia; ^12^ Centre for Epidemiology and Biostatistics, School of Population and Global Health, University of Melbourne Melbourne Victoria Australia

**Keywords:** breast cancer, co‐production, intervention, mammography, obesity, participation, rescreening, screening

## Abstract

**Introduction:**

Higher BMI is associated with reduced breast screening participation but it is unclear if BMI is a proxy for other potential barriers to screening. This study describes the correlations between BMI and body image, perceptions of risk, and pain in a large sample of breast screening participants to help identify targets for improving screening participation within people with obesity.

**Methods:**

A survey collected information on body image, perceived risk of breast cancer, and pain experienced during mammography from 10,751 participants attending BreastScreen Western Australia between June and December, 2023. Latent variable analysis was used to generate single unobserved index variables for perceived risk, body surveillance, and body shame. Spearman's rho correlations were estimated between BMI, four body shame variables, six body surveillance variables, overall perceived risk index, and a pain score.

**Results:**

BMI was significantly positively correlated with responses indicating increased body shame and increased body surveillance, except for one question where participants with increased BMI indicated that they think it is more important that their clothes are comfortable than look good. BMI was also positively correlated with overall perceived risk index (*r*_*s*_ = 0.08, *p* < 0.0001), and negatively correlated with the pain score (*r*_*s*_ = −0.04, *p* < 0.0001).

**Conclusion:**

The findings demonstrate that BMI is more highly correlated with body shame than body surveillance, perceived risk, or breast pain. Targeting body shame barriers to screening may improve participation within people with obesity, thereby improving breast cancer outcomes in this high‐risk group.

## 1 Introduction

Globally, female breast cancer incidence is increasing [1]. Mammography screening participation is essential for early cancer detection and improved breast cancer outcomes [2]. Participation rates in Australia are lower than the recommended standard of ≥ 70% for effective early breast cancer detection, pre‐ and post‐COVID pandemic (~50%–55%) [3, 4]. Body mass index (BMI) is negatively associated with screening participation and women with obesity (BMI ≥ 30 kg/m^2^) are less likely to return to screening when next due [5]. Screening is particularly important for women with obesity as increased BMI is associated with an increased risk of postmenopausal breast cancer [6], poorer prognosis [7], and higher breast cancer mortality rates [8]. BMI is often considered a barrier to screening participation, but it's unclear if it's a proxy for other potential drivers of lower mammography participation such as body image concerns [9], pain [10], and perceived risk [11, 12, 13]. However, it is not known to what extent these factors are correlated with BMI. Increased understanding of the relationship between BMI and these barriers to breast cancer screening is important to inform targeted strategies to improve screening participation, particularly within women with obesity.

Body image is a multidimensional concept involving positive and negative self‐perceptions and attitudes towards the body [14, 15]. Women with obesity or who are overweight often experience more body image disturbances or shame [15]. Mammography requires partial undressing, from the waist up, which may increase self‐consciousness or feelings of vulnerability among women with obesity or who are overweight. This can lead to increased body image concerns, including the fear of judgement from screening staff, possibly resulting in non‐reattendance to breast screening [9]. Moreover, body image is a subjective construct shaped by social influences, with Western ideals promoting slenderness and associating it with self‐discipline, leading to stigma for those who do not conform [16]. Studies indicate that higher BMI is negatively correlated with body image appreciation, especially among women with obesity or who are overweight [14, 15, 16]. Some of the reasons women with high body shame issues do not attend breast screening include embarrassment and discomfort with their bodies and/or breasts [17, 18]. The relationship between BMI and body image is crucial as it influences women's comfort during procedures requiring partial nudity.

Women with obesity may also face positioning challenges, including extra imaging views to demonstrate all of the breast tissue adequately, skin folds and splitting, or mobility limitations [19]. These challenges can increase pain/discomfort during the procedure and may lower client satisfaction with screening [20]. A systematic review reported that 25%–46% of women did not reattend mammography screening due to pain [10]. Other studies found that pain during mammography increases with higher BMI [21, 22] and can also be influenced by radiographer‐client communication, equipment, and the specific context [22]. Despite the association of BMI and pain and its impact on screening reattendance, limited data exist, indicating the need for further studies.

Finally, there is limited information regarding how women with obesity perceive their risk of breast cancer. Studies indicate that women aged 18+ with obesity or who are overweight perceive their breast cancer risk similarly to those in other BMI categories, despite a majority recognising the relationship between obesity and increased breast cancer risk [11, 12]. However, reduced perceived breast cancer risk among women with obesity may discourage them from participating in screening, as they may not recognise their status as high‐risk individuals [13]. Further studies are needed to inform how women with obesity perceive and manage their individual risk (e.g., screening participation).

The present study is part of the BreastScreenPlus project which aims to co‐produce a novel, two‐part intervention comprising a client‐focused component and a staff‐focused component to improve screening participation. Within a large cross‐sectional survey of women attending population‐based mammography screening, this current study describes the relationship of BMI with body image, perceptions of risk, and pain. Results will contribute to a more nuanced understanding of how BMI may impact the mammogram experience and subsequent rescreening via investigations of BMI's relationship with other related barriers to screening.

## 2 Methods

### 2.1 Recruitment

All clients attending screening at BreastScreen Western Australia from 21 June 2023 to 20 December 2023 were invited to complete a survey via email after their routine screening mammogram. Responses were anonymous unless women opted to provide their contact details for future research opportunities.

### 2.2 Survey Development

Survey questions were identified from existing literature and adapted from a comprehensive literature review to assess participant perceptions of their body image [23], breast cancer risk [24], and pain during mammography [22]. The adapted questions were reviewed and shortlisted by the BreastScreenPlus Stakeholder Consultant Group. The Objectified Body Consciousness Scale (OBCS) was identified as the best measure of body image based on its validity, reliability, and suitability to the target population and purpose of this study [23]. The OBCS has three subscales including body surveillance, body shame, and appearance control beliefs with 24 questions in total [23]. The survey was designed to include ten of the OBCS possible items from the body surveillance and body shame subscales to ensure appropriateness and brevity of survey questions [23]. The body surveillance subscale included 6 questions on how women perceive themselves in relation to how others see them (public self‐consciousness). The body shame subscale consisted of four questions assessing a woman's internalisation of cultural body standards by measuring the shame she feels when she perceives herself as not meeting these ideals. Participants rated the ten body surveillance and body shame questions on a 5‐point Likert scale from strongly disagree to strongly agree. The questions were a combination of positive and negative worded statements to control for acquiescence bias (Supporting Information S1). Breast cancer risk perception was assessed using three questions, while pain during mammography was measured on a scale ranging from 0 to 100 (Appendix S1).

The survey also collected basic demographic information including age, height, weight, postcode, country of birth, and language spoken other than English at home. BMI was calculated using self‐reported height and weight measurements and categorised into five groups: underweight and healthy weight (< 18.5–25 kg/m^2^), overweight (25–30 kg/m^2^), obese I (30–35 kg/m^2^), obese II (35–40 kg/m^2^), and obese III (> 40 kg/m^2^). Country of birth was categorised into four groups: Australia/Europe/United Kingdom, South Pacific/New Zealand, Asia, and Other. Individuals were assigned Accessibility/Remoteness Index for Areas (ARIA) [25] and Socio‐Economic Indexes for Areas (SEIFA) [25] from Australian Bureau of Statistic's 2021 census data based on their residential postcode. Remoteness was categorised into four groups: major city, inner regional, outer regional, and remote/very remote. The study employed quintiles of SEIFA's Index of Relative Socio‐economic Disadvantage where the first quintile indicates the most disadvantaged population [25]. Survey data was collected using the Qualtrics software program.

### 2.3 Exclusion and Inclusion Criteria

Participants who provided their height and weight measurements were included in this study. Participants with questionable measurements of height, weight and BMI were excluded (height < 1 m and > 2 m, weight < 25 kg and > 220 kg and BMI > 70 kg/m2).

### 2.4 Statistical Analysis

#### 2.4.1 Latent Variable Modelling

Based on previous work [24], the three observed perceived risk questions were combined using latent variable modelling to estimate an unobserved overall perceived risk index. A substantial proportion of survey respondents (20.3%) provided an exact 50% response to the question regarding the chance of developing breast cancer in their lifetime on a scale from 0 to 100, indicating ambiguity or dichotomy in a breast cancer diagnosis. Quintile cut‐points were used to convert this numeric variable into an ordinal variable, omitting values of precisely 50%. Graded response models (GRMs) were used to model manifest variables. The overall perceived risk index was estimated twice—fitting GRMs with and without allowance for those who responded precisely 50% [24]. Model fit diagnostics (residual plots, marginal χ^2^ contingency table tests of model fit, item response curves, coefficient estimates) were evaluated for both GRMs. The factor scores for each response pattern were then rescaled and mapped back to the respondent data to obtain a perceived risk index.

The same latent variable modelling method was applied separately to the six observed body surveillance and the four body shame variables to estimate manifest variables for an (unobserved) body surveillance index and body shame index. To ensure the uniformity of the Likert scale, negatively worded statements were converted to positive ones by reverse coding before calculating the overall index.

#### 2.4.2 Descriptive Analyses

Mann–Whitney *U* tests were used to compare the demographic characteristics between included and excluded participants due to missing BMI, including technical errors within the Qualtrics survey.

Questionnaire responses measured using the 5‐point Likert scale were analysed as ordinal measures. Spearman's rho correlation was calculated to describe the relationship between the variables.

Spearman's rho ranges from −1 (perfect negative) to 0 (no relationship) to +1 (perfect positive).

Data cleaning was conducted in SPSS version 29 and all other analyses were completed in R version 4.4.1 [26].

## 3 Results

**FIGURE 1:**
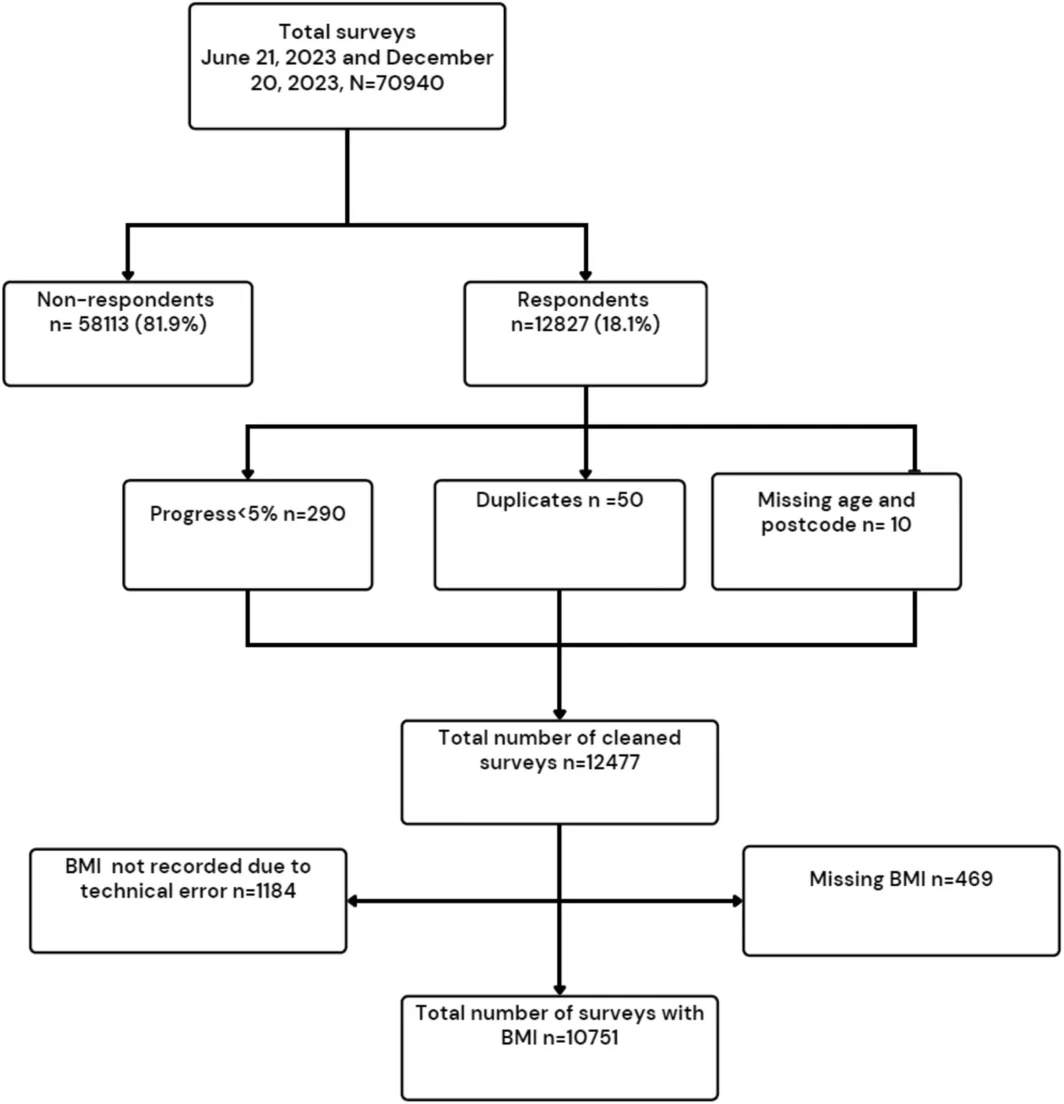
Flowchart of the exclusion criteria of the study participants attending BreastScreen Western Australia between June–December 2023.

The survey received an 18% response rate, with 12,827 out of 70,940 participants responding (Figure 1). Among those, 10,751 provided their height and weight measurements. Due to technical errors within the Qualtrics survey, some participants were unable to enter their height and weight measurements; as a result, they were excluded from the analysis (*n* = 1184). The technical error was randomly distributed across participants; however, there was evidence of small statistical differences in age and SEIFA between those excluded and included in the study; primarily they were older with lower socioeconomic index compared to those included (Appendix S2). The data exclusion procedure is outlined in Figure 1. The final sample size was 10,751.

The characteristics of participants are presented in Table 1. The largest proportion of participants fell within the age group 60–64 years (19.3%). Participants were predominantly born in Australia/Europe/United Kingdom (61.6%), spoke English at home (91.7%), resided in major cities (77.9%), and in higher quintiles of the socio‐economic indices (27.7%).

**TABLE 1:** Characteristics of mammographic screening events at BreastScreen Western Australia between June 2023–December 2023.

Characteristics	Response	Frequency (%)
Age at survey	40–44 years	775 (7.2)
	45–49 years	898 (8.4)
	50–54 years	1484 (13.8)
	55–59 years	1788 (16.6)
	60–64 years	2077 (19.3)
	65–69 years	1872 (17.4)
	70–74 years	1425 (13.3)
	75–79 years	344 (3.2)
	80+ years	88 (0.8)
BMI^a^ Categories	Underweight and Healthy weight (< 18.5 –25 kg/m^2^)	3672 (34.2)
	Overweight (25–30 kg/m^2^)	3659 (34.0)
	Obese I (30–35 kg/m^2^)	2061 (19.2)
	Obese II (35–40 kg/m^2^)	870 (8.1)
	Obese III (> 40 kg/m^2^)	489 (4.5)
	Missing	0
ARIA^b^	Major city	8379 (77.9)
	Inner regional	1027 (9.6)
	Outer regional	886 (8.2)
	Remote & Very remote	408 (3.8)
	Missing	51 (0.5)
Relative socio‐economic disadvantage index^c^	1 (lowest)	1160 (10.8)
	2	2142 (19.8)
	3	2088 (19.4)
	4	2313 (21.5)
	5 (highest)	2978 (27.7)
	Missing	70 (0.7)
Country of birth	Australia, Europe, and United Kingdom	9415 (87.8)
	South Pacific and New Zealand	391 (3.6)
	Asia	505 (4.7)
	Other	416 (3.9)
Any language spoken other than English at home	Yes	881 (8.2)
	No	9862 (91.7)
	Missing	8 (0.1)

^a^ BMI is body mass index.

^b^ Accessibility/Remoteness Index of Australia (ARIA) scores.

^c^ Relative socio‐economic disadvantage index (SEIFA) is on a scale from 1 to 5, where 1 indicates the lowest 20% of the population in Western Australia (few households with high incomes, few people with qualifications, few people in high‐skilled occupations) and 5 indicates the highest 20% of the population in Western Australia (most households with high incomes, most people with qualifications, most people in high‐skilled occupations).

The distributions of each of the outcome variables are provided in Figure S3. Median scores of 11 ± 37 and 30.19 ± 27.25 were recorded for pain scores and overall perceived risk index respectively. The most common responses in body image subscales were “agree” and “disagree” indicating a strong dichotomy of opinion. For example, for the body shame subscale statement, “I feel embarrassed of myself when I haven't made an effort to look my best”, 38% agreed whilst 40% disagreed. Similarly, for the body surveillance subscale statement “I rarely compare how I look with how other people look” 40% disagreed while 37% agreed. More than half of participants disagreed with statements regarding body surveillance, with 59% disagreeing on thinking rarely about appearance and 54% thinking about their looks frequently. Conversely, fewer participants disagreed with the importance of comfort over appearance in clothing (31%) and prioritising how their body feels over how it looks (24%). More than half of the participants disagreed with the body surveillance statements “I rarely think about how I look” (59%) and “During the day, I think about how I look many times” (54%). In contrast, fewer participants disagreed with the importance of comfort over appearance in clothing (31%) and prioritising how their body feels over how it looks (24%) (Appendix S3).

Model fit diagnostics for the latent variable analyses are presented in Appendix S4. For the three perceived risk questions, the GRM which excluded 50% responses from mean and standard deviation calculations demonstrated a good fit and was used in further analyses as a latent variable representing overall perceived risk. The body surveillance and body shame models did not demonstrate a good fit for the latent variable modelling and therefore were not used in further analyses (Appendix S4). Rather, the raw values of the responses to each question were used.

**FIGURE 2:**
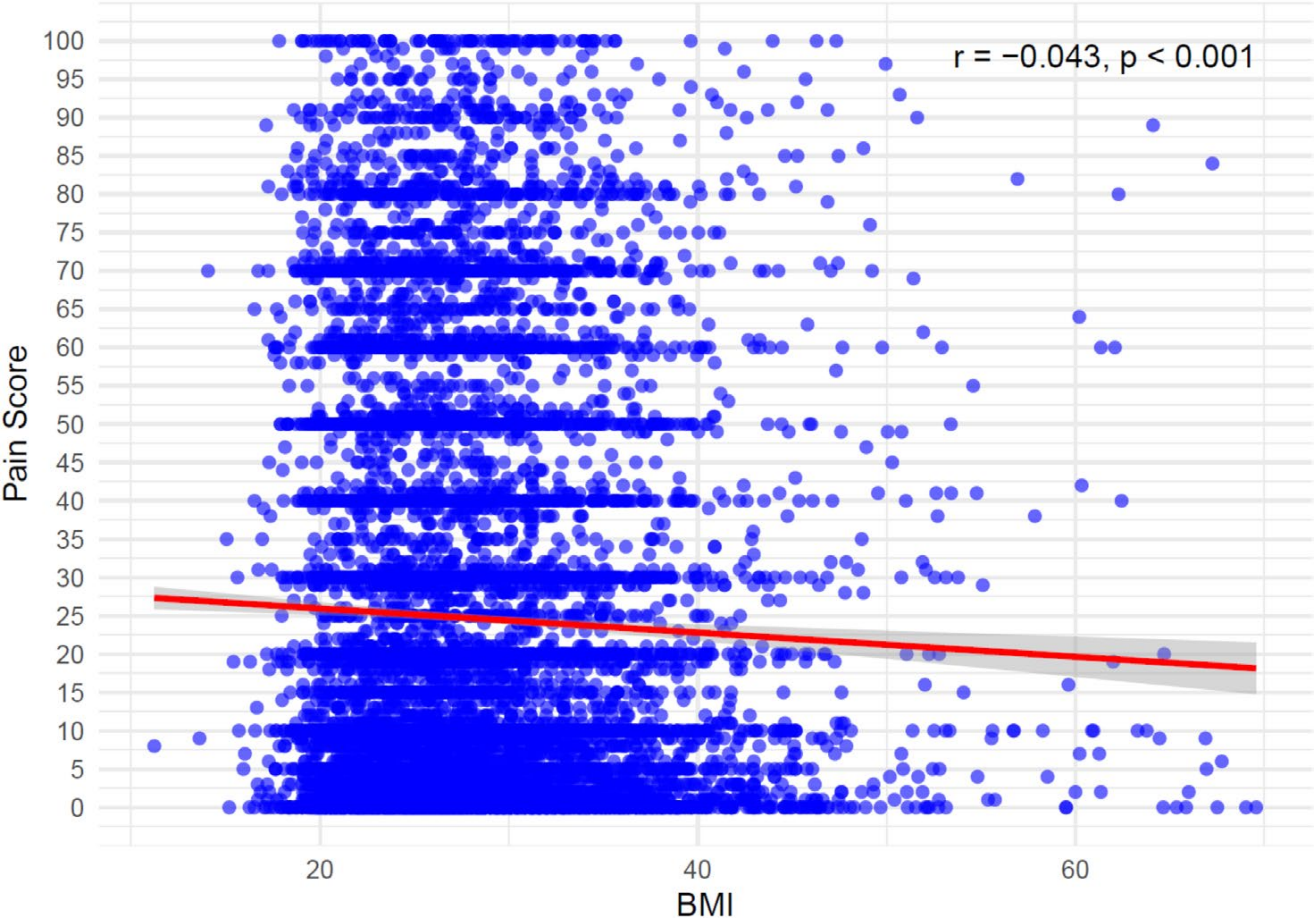
Scatterplot of self‐reported level of pain score experienced during mammography by BMI (kg/m^2^). Scatterplot of the relationship between pain score and BMI for 10,751 participants attending BreastScreen Western Australia between June to December 2023. Each blue dot represents an individual participant's response. BMI is in kg/m^2^; *r* = −0.043 is the Spearman rho correlation with *p*‐value < 0.001.

**FIGURE 3:**
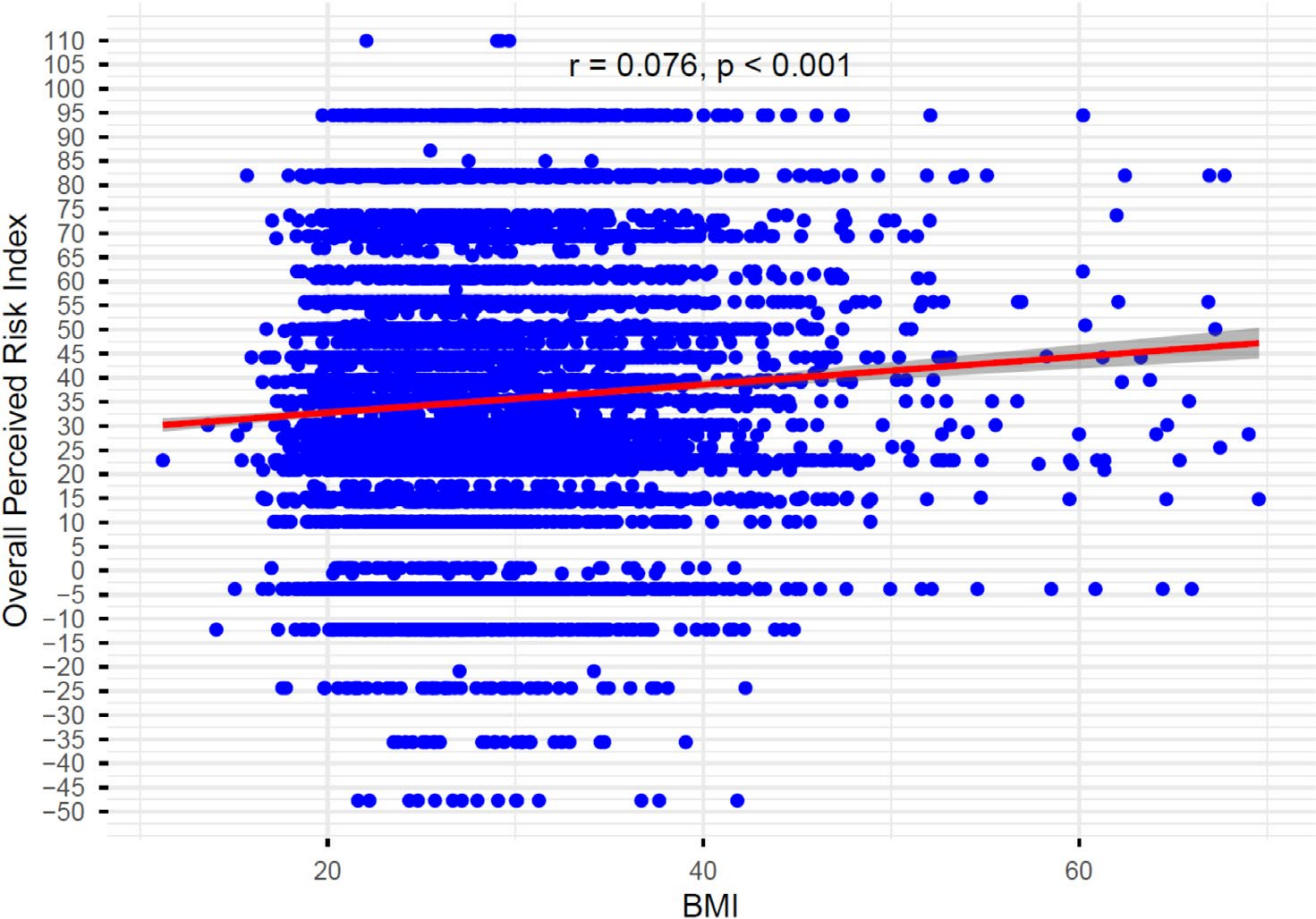
Scatterplot of Overall Perceived Risk Index by BMI (kg/m^2^). Scatterplot of the relationship between overall perceived risk index and BMI for 10,751 participants attending BreastScreen Western Australia between June to December 2023. Each blue dot represents an individual participant's response. BMI is in kg/m^2^; *r* = 0.076 is the Spearman rho correlation with *p*‐value < 0.001.

**FIGURE 4:**
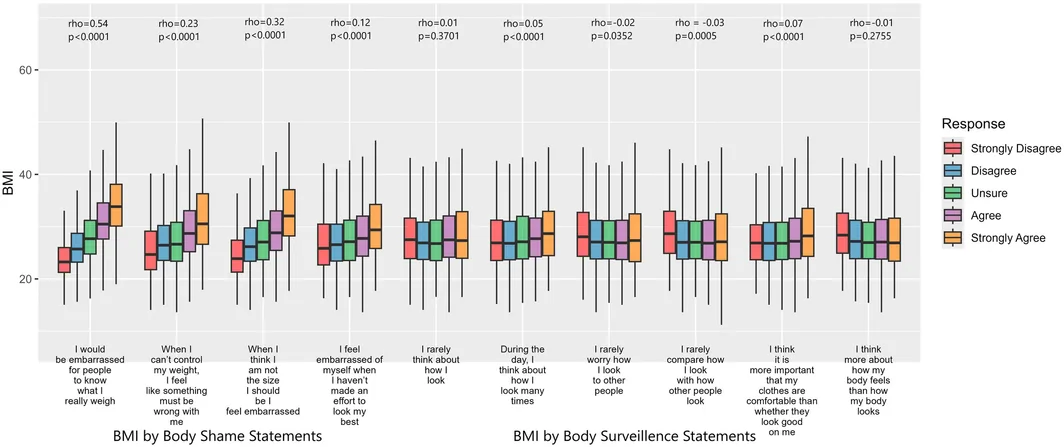
Responses to body image questions stratified by BMI (kg/m^2^). Boxplots of the relationship between BMI and the ten body image statements for 10,751 participants attending BreastScreen Western Australia between June and December 2023. The first four statements from the left are body shame statements, and the remaining six are body surveillance statements. The boxplot is complemented by Spearman's rho correlation (rho) with *p*‐values (*p*).

**FIGURE 5:**
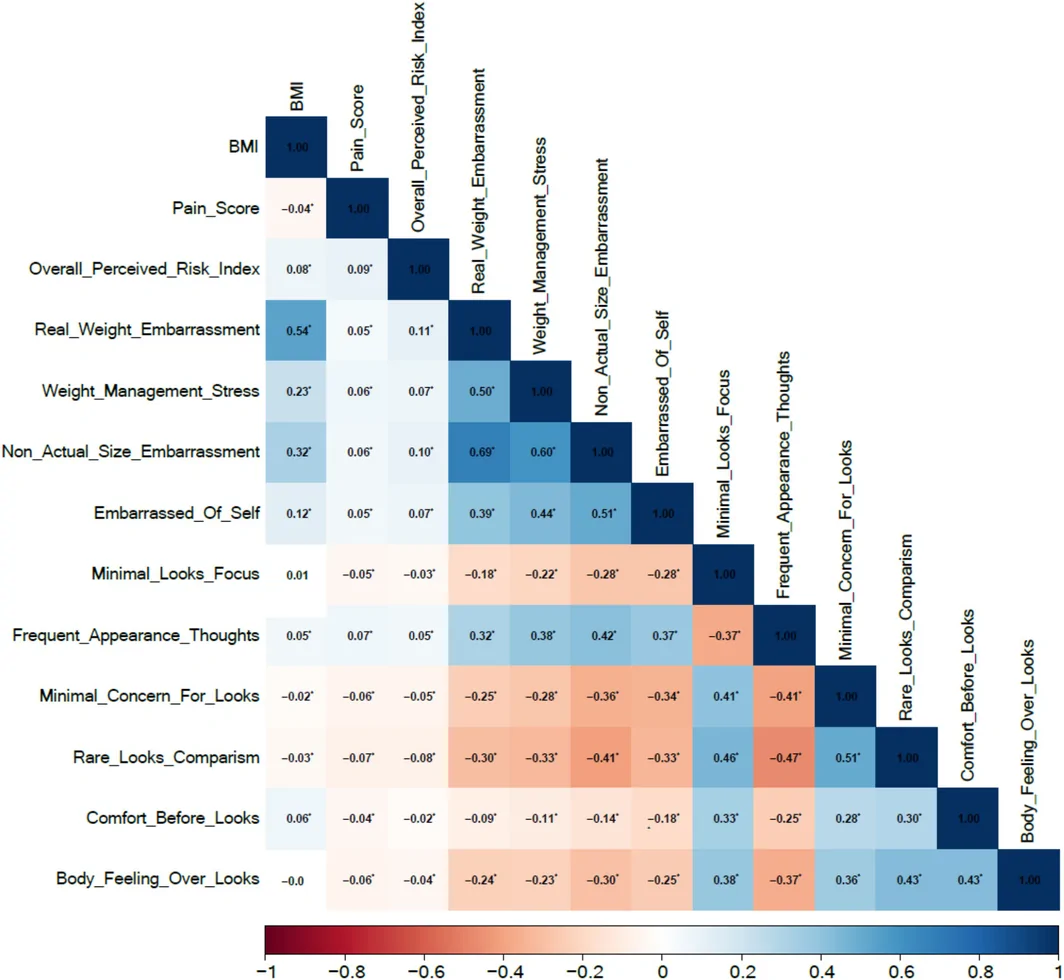
Heatmap of Spearman's Rho correlation matrix for the study variables. The heatmap displays Spearman rho correlation between BMI, pain scores, and ten body image statements for 10,751 participants attending BreastScreen Western Australia between June–December 2023. Coefficients are displayed inside each cell with deeper colours representing stronger correlations. Only significant correlations are represented with asterisks (*) of *p* value < 0.05. BMI , body mass index; Body_Feeling_Over_Looks = I think more about how my body feels than how my body looks; Frequent_Appearance_Thoughts, During the day, I think about how I look many times; Minimal_Concern_for_Looks, I rarely worry how I look to other people; Non‐Actual_Size_Embarrassment, When I think I am not the size I should be, I feel embarrassed; Embarrassed_of_Self, I feel embarrassed of myself when I haven't made an effort to look my best; Minimal_Looks_Focus, I rarely think about how I look; Rare_Looks_Comparism, I rarely compare how I look with how other people look. Comfort_Before_Looks = I think it is more important that my clothes are comfortable than whether they look good on me; Real_Weight_Embarrassment, I would be embarrassed for people to know what I really weigh; Weight_Management_Stress, When I can't control my weight, I feel like something must be wrong with me.

There was a significant negative correlation between BMI and the pain score (Figure 2) and a significant positive correlation with the perceived risk latent variable (Figure 3). Eight of the ten body image statements were significantly correlated with BMI (*p* < 0.05, Figure 4). There was a clear upward trend (*p* < 0.0001) within three of the body shame statements, with median BMI increasing progressively from strongly disagree to strongly agree. These were: “I would be embarrassed for people to know what I really weigh” (*r*_*s*_ = 0.54, *p* < 0.0001), “When I can't control my weight, I feel something must be wrong with me” (*r*_*s*_ = 0.23, *p* < 0.0001), and “When I think I am not the size I should be, I feel embarrassed” (*r*_*s*_ = 0.32, *p* < 0.0001) shown in Figure 5 and Appendix S5.

In contrast, the six body surveillance statements showed a less consistent relationship with BMI, although four of the six statements were still significantly correlated with BMI. BMI showed a very weak positive correlation with “During the day, I think about how I look many times” (*r*_*s*_ = 0.05, *p* < 0.0001), and “I think it is more important that my clothes are comfortable than whether they look good on me” (*r*_*s*_ = 0.07, *p* < 0.0001) and a weaker negative correlation with the statements, “I rarely worry how I look to other people” (*r*_*s*_ = −0.02, *p* = 0.0352) and “I rarely compare how I look with how other people look” (*r*_*s*_ = −0.03, *p* = 0.0005).

Most of the body shame and body surveillance statements showed moderate/strong correlation with each other (Figure 5 and Appendix S5), especially three body shame statements which are highly correlated with BMI. Pain score showed low positive correlation with the overall perceived risk index (*r*_*s*_ = 0.09, *p* < 0.0001) and low negative correlation with BMI (*r*_*s*_ = −0.04, *p* < 0.0001). Similarly, the overall perceived risk index was very weakly positively correlated with BMI (*r*_*s*_ = 0.08, *p* < 0.0001) (Figure 5 and Appendix S5).

## 4 Discussion

The current study investigated the relationship of BMI with body image, perceptions of risk, and pain. BMI showed a weak negative correlation with pain and a weak positive correlation with perception of risk. BMI showed strong positive correlations with body shame and weaker positive correlations with body surveillance. Based on these results, targeting strategies to address body shame issues may yield the best improvements in screening rates among people with obesity rather than focusing on body surveillance, pain, and perceptions of risk.

The findings show that of all the factors considered, body shame has the highest correlation with BMI and may be a driver of lower mammographic participation among women with obesity. This is consistent with a previous study [15], indicating that women with higher BMI experience higher body shame. This implies that women with obesity may internalise cultural ideals and perceive slenderness as the standard body type. Another study suggests that women with high body shame may feel uncomfortable with their bodies and breasts, leading them to avoid participating in breast screening programs [17]. This study found the strongest correlation between BMI and the statement “I would be embarrassed for people to know what I really weigh”, indicating that discomfort with being weighed may be impacting participants' willingness to rescreen. In addition, women with high body shame may perceive themselves as morally and physically inferior due to their physical traits, internalising beliefs that losing control over their body is shameful, as societally it may be seen as indicative of laziness or lack of will power in cultural messages [18]. The findings of the present study support this, as participants with higher BMI felt something must be wrong with them when they could not control their weight. Since breast screening involves some nudity, participants with obesity who experience body shame may feel too ashamed to participate as they do not meet cultural expectations of their bodies.

The current study found a weaker but significant correlation between increasing BMI and increased body surveillance. Previous, smaller studies [27, 28] have also reported a weak positive correlation between BMI and body surveillance, although these correlations were not significant, possibly due to reduced power or differences in population characteristics, including relatively younger age distribution and lower BMI levels.

The moderate to strong correlations observed among body shame statements suggest they capture a more coherent and internally consistent construct, compared to weak to moderate correlations among the body surveillance statements. Despite the correlations observed, the latent variable for body surveillance and body shame still did not demonstrate a good fit. This suggests that, while the body image statements appear conceptually related and internally consistent, they could not fully capture a unidimensional construct when modelled as a latent variable, possibly due to redundancy.

The overall perceived risk index demonstrated a very weak positive significant correlation with BMI, in contrast with previous studies [11, 12] which reported no difference in the perceived risk of breast cancer among the various BMI categories. Methodological differences could be the cause of this discrepancy, and this study's larger sample size could help to explain why a weak effect became significant. Overall, these findings suggest that more education may be warranted to inform women with obesity of their increased risk of postmenopausal breast cancer [6]. However, based on the findings of the current study, the perception of breast cancer risk is unlikely to explain the reduced participation in women with obesity.

Additionally, this present study found a very weak negative correlation between BMI and pain score, contradicting previous findings [21, 22] which reported increased pain during mammography for women with obesity. A woman's pain during mammography is influenced by factors such as breast size, density, and positioning, which can impact the distribution and amount of compression forces applied [21, 22]. The current study's pain levels differed from previous studies possibly due to this study being a single scale measurement, contrasting with detailed clinical factors assessed in previous studies. For example, Moshina et al. [22] reported that compressed breast thickness was higher for women who experienced strong pain and was partly influenced by the compressibility of their breasts. The authors suggested the higher pain experienced by women with obesity could be due to the low compressibility of their breasts, making them more sensitive to pain [22]. The low pain levels reported by participants with obesity in this current study indicate that pain is also unlikely to explain low screening participation in people with obesity.

### 4.1 Limitations of This Study

The use of self‐reported body weight and height to calculate BMI could have introduced measurement bias. The exclusion of a subset of the data due to technical error reduced the statistical power of the study and may have introduced some selection bias. The study sample only included women who attended the population‐based breast screening program; therefore, women who are unwilling to screen may have been excluded from the study.

## 5 Conclusion

The findings of this study demonstrate high correlations between measures of body shame and BMI, and much more modest correlations between BMI and measures of body surveillance, perceptions of breast cancer risk, and pain scores in a sample of women who had attended population‐based breast screening. This study therefore proposes that body shame may partially explain reduced screening participation observed in women with obesity. This will be explored in the full analysis of rescreening as part of the BreastScreenPlus project to determine the levels of satisfaction with screening and intention to rescreen among women with obesity pre and post intervention. The current study results indicate that the development of tailored strategies or interventions to address body shame issues during screening may improve screening participation, particularly for women with obesity. This could in turn lead to early breast cancer detection and improved survival within this higher‐risk group.

## Author Contributions

**Elizabeth Dzidzornu:** conceptualization, methodology, software, data curation, formal analysis, writing – original draft, writing – review and editing, resources, investigation, validation, visualization.

## Funding

This work was funded by the Australian National Health and Medical Research Council [APP2015286]. J.S. is supported by Cancer Council Western Australia.

## Ethics Statement

Ethical approval was obtained from the Western Australian Department of Health Human Research Ethics Committee (RGS0000005946) and the University of Western Australia Human Research and Ethics Committee (2022/ET000593).

## Conflicts of Interest

The authors declare no conflicts of interest.

## Supporting information


**Appendix S1:** BreastScreen WA Client Survey.
**Appendix S2:** Frequency of characteristics of participants excluded from the analyses compared to those included.
**Appendix S3a:** Frequency of responses to ten body image statements.
**Appendix S3b:** Histogram of self‐reported level of pain score experienced during mammography.
**Appendix S3c:** Histogram of responses of Overall perceived risk index.
**Appendix S3:** Frequency of responses to ten body image statements (a) histogram of self‐reported level of pain score experienced during mammography (b) and histogram of responses of Overall perceived risk index (c) for 10,751 participants attending BreastScreen Western Australia between June to December 2023. Participants rated each of the ten body surveillance and body shame questions on a 5‐point Likert scale from strongly disagree to strongly agree. The median and interquartile range are shown for each histogram.
**Appendix S4a:** Perceived Risk Latent Variable Model Analyses 1.
**Appendix S4b:** Perceived Risk Latent Variable Model Analyses 2.
**Appendix S4c:** Body Shame Latent Variable (5 responses) Model Analyses.
**Appendix S4d:** Body Surveillance Latent Variable (5 responses) Model Analyses.
**Appendix S4e:** Body Shame Latent Variable (3 responses) Model Analyses.
**Appendix S4f:** Body Surveillance Latent Variable (3 responses) Model Analyses.
**Appendix S4:** Chi‐square residuals with factor scores for modelled response patterns by Graded Response Models (GRMs). Plots represent GRM fits to (a) all 3 ordinal perceived risk variables (manifest variables) for all individuals (a) and separately to data for individuals: (b) providing a Numeric score of exactly 50%, with Verbal and Comparative as the manifest variables (GRM 2a); and (c) providing a Numeric score other than exactly 50%, with all 3 perceived risk variables as the manifest variables (GRM 2b). Green dashed lines show fitted local regressions demonstrating the trend in residuals, as distributed with model‐estimated values of the latent trait. For GRM 2b, the x‐axis scale has been reversed to ensure that factor scores are comparable with those estimated for GRM 1 and GRM 2a, in terms of the unobserved overall perceived risk. Similarly, chi‐square residuals with factor scores for modelled response patterns by GRMs for an (unobserved) body shame index and body surveillance index using the 5‐point Likert scale or following conversion to three responses are shown in,d,e,f.
**Appendix S5:** Spearman's Rho Correlation Matrix for the study variables.

## Data Availability

The data that support the findings of this study are available from the corresponding author upon reasonable request.
