# Supplementary material for: Correlation of BMI With Potential Barriers to Mammographic Screening: Body Image, Pain, and Perceived Risk of Breast Cancer–A Cross‐Sectional Study
Source: J Med Imaging Radiat Oncol. 2026 Mar 19;70(6):495–504. doi: 10.1111/1754-9485.70085 (PMC13580417; doi:10.1111/1754-9485.70085)
Supplement: Supplementary file 1 [file ARA-70-495-s001.docx]

# Supplementary Material 1: BreastScreen WA Client Survey

Thank you for participating in this survey. It should only take 5-10 minutes to complete depending on your responses. Please read the Participant Information and Statement of Consent sheet. By participating in the survey you are consenting to the terms outlined in this sheet.

Please do not forward the link to this survey to non-invited participants.

We thank you for considering participating in this important research.

## 1.0 Introduction.

1. What is your age?

□ 40 – 44 years

□ 45 – 49 years

□ 50 – 54 years

□ 55 – 59 years

□ 60 – 64 years

□ 65 – 69 years

□ 70 – 74 years

□ 75 – 79 years

□ 80+ years

1.1 What is your postcode? ______________________

## 2. Pain

On a scale from 0 to 10, please score your level of pain experienced during your mammogram (where 0 indicates no pain and 10 indicates very strong pain)

No pain 0	1	2	3	4	5	6	7	8	9	10 Very Strong Pain

## 3.0 Body Image

We would now like to ask you about how you feel about your body. Please indicate how much you agree with each of the following statements using the below scale.

□ Strongly Disagree	□ Disagree	□ Unsure	□ Agree	□ Strongly Agree

1. I rarely think about how I look

□ Strongly Disagree	□ Disagree	□ Unsure	□ Agree	□ Strongly Agree

2. I think it is more important that my clothes are comfortable than whether they look good on me

□ Strongly Disagree	□ Disagree	□ Unsure	□ Agree	□ Strongly Agree

3. I think more about how my body feels than how my body looks

□ Strongly Disagree	□ Disagree	□ Unsure	□ Agree	□ Strongly Agree

4. I rarely compare how I look with how other people look

□ Strongly Disagree	□ Disagree	□ Unsure	□ Agree	□ Strongly Agree

5. During the day, I think about how I look many times.

□ Strongly Disagree	□ Disagree	□ Unsure	□ Agree	□ Strongly Agree

6. I rarely worry how I look to other people – ---

□ Strongly Disagree	□ Disagree	□ Unsure	□ Agree	□ Strongly Agree

7. When I can’t control my weight, I feel like something must be wrong with me

□ Strongly Disagree	□ Disagree	□ Unsure	□ Agree	□ Strongly Agree

8. I feel embarrassed of myself when I haven’t made an effort to look my best

□ Strongly Disagree	□ Disagree	□ Unsure	□ Agree	□ Strongly Agree

9. I would be embarrassed for people to know what I really weigh

□ Strongly Disagree	□ Disagree	□ Unsure	□ Agree	□ Strongly Agree

10. When I think I am not the size I should be I feel embarrassed

□ Strongly Disagree	□ Disagree	□ Unsure	□ Agree	□ Strongly Agree

## 4.0 Perceptions and Attitude

The following questions relate to your personal awareness and attitudes towards the development of breast cancer.

1. What do you think your chance is of developing breast cancer in your lifetime? Please choose a number between 0% (no chance of breast cancer) and 100% (definitely will get breast cancer) ______________

2. How would you rate your chance of developing breast cancer?

□ Very low	□ Moderately low	□ Neither high nor low	□ Moderately high	□ Very high

3. Overall, how do you think your chance of developing breast cancer compares to the average woman your age? Please circle below.

□ Less likely	□ About as likely	□ More likely

## 5.0 Demographic Information

1. What is your weight (kgs)? ___________

2. What is your height (cm)? ____________

3. What is your country of birth? ___________________

4. Do you speak a language OTHER than English at home? ________

4.1 If YES, what language? _________________________________

# Supplementary Material 2 _ Frequency of characteristics of participants excluded from the analyses compared to those included

| **Characteristics** | **Response** | **Frequency (%)** | | | **Mann-Whitney U Test** | |
| --- | --- | --- | --- | --- | --- | --- |
|  |  | **Participants with BMI**  **[n=10751 (100%)]** | **Participants with Technical Error BMI [n = 1184 (100%)]** | **Participants with Missing BMI**  **[n = 469 (100%)]** | **Participants with BMI**  **& Participants with Technical Error BMI** | **Participants with BMI**  **& Participants with Missing BMI** |
| Age at survey [n(%)] | 40 – 44 years | 775 (7.2) | 42 (3.5) | 25 (5.3) | <0.001 | <0.001 |
|  | 45 – 49 years | 898 (8.4) | 49 (4.1) | 26 (5.5) |  |  |
|  | 50 – 54 years | 1484 (13.8) | 79 (6.7) | 50 (10.7) |  |  |
|  | 55 – 59 years | 1788 (16.6) | 147 (12.4) | 76 (16.2) |  |  |
|  | 60 – 64 years | 2077 (19.3) | 198 (16.7) | 109 (23.2) |  |  |
|  | 65 – 69 years | 1872 (17.4) | 256 (21.6) | 73 (15.6) |  |  |
|  | 70 – 74 years | 1425 (13.3) | 270 (22.8) | 79 (16.8) |  |  |
|  | 75 – 79 years | 344 (3.2) | 96 (8.1) | 25 (5.3) |  |  |
|  | 80+ years | 88 (0.8) | 47 (4.0) | 6 (1.3) |  |  |
|  |  |  |  |  |  |  |
| ARIA ª [n(%)] | Major city | 8379 (77.9) | 904 (76.4) | 344 (73.3) | 0.172 | 0.012 |
|  | Inner regional | 1027 (9.6) | 118 (10.0) | 52 (11.1) |  |  |
|  | Outer regional | 886 (8.2) | 104 (8.8) | 42 (9.0) |  |  |
|  | Remote & Very Remote | 408 (3.8) | 34 (2.9) | 16 (3.4) |  |  |
|  | Missing | 51 (0.5) | 24 (2.0) | 15 93.2) |  |  |
|  |  |  |  |  |  |  |
| Relative socio-economic disadvantage index ^b^ | 1(lowest) | 1160 (10.8) | 176 (14.9) | 68 (14.5) | <0.001 | 0.006 |
|  | 2 | 2142 (19.8) | 250 (21.1) | 99 (21.1) |  |  |
|  | 3 | 2088 (19.4) | 251 (21.2) | 107 (22.8) |  |  |
|  | 4 | 2313 (21.5) | 218 (18.4) | 78 (16.6) |  |  |
|  | 5 (highest) | 2978 (27.7) | 262 (22.1) | 101 (21.5) |  |  |
|  | Missing | 70 (0.7) | 27 (2.3) | 16 (3.4) |  |  |
|  |  |  |  |  |  |  |
| Country of birth [n(%)] | Australia, Europe, and United Kingdom | 9415 (87.8) | - | 351 (74.9) | - | 0.250 |
|  | South Pacific and New Zealand | 391 (3.6) | - | 15 (3.2) |  |  |
|  | Asia | 505 (4.7) | - | 11 (2.3) |  |  |
|  | Other | 416 (3.9) | - | 10 (2.2) |  |  |
|  |  |  |  |  |  |  |
| Any language spoken other than English at home [n(%)] | Yes | 881 (8.2) | - | 35 (7.5) | - | <0.001 |
|  | No | 9862 (91.7) | - | 390 (83.2) |  |  |
|  | Missing | 8 (0.1) | - | 44 (9.4) |  |  |

^a^ Accessibility/Remoteness Index of Australia (ARIA) scores

^b^ Relative socio-economic disadvantage index is on a scale from 1 to 5, where 1 indicates the lowest 20% of the population in Western Australia (few households with high incomes, few people with qualifications, few people in high skilled occupations) and 5 indicates the highest 20% of the population in Western Australia (most households with high incomes, most people with qualifications, most people in high skilled occupations)

BMI is body mass index of all clients who recorded their height and weight.

Technical Error BMI is body mass index of all clients who could not record their height and weight due to technical challenges.

Missing BMI is body mass index of all clients who voluntarily did not record their height and weight.

# Supplementary Figure S3a: Frequency of responses to ten body image statements

Body Image Statements

# Supplementary Figure S3b: Histogram of self-reported level of pain score experienced during mammography

# Supplementary Figure S3c: Histogram of responses of Overall perceived risk index

**Supplementary Figure S3:** Frequency of responses to ten body image statements (S3a), histogram of self-reported level of pain score experienced during mammography (S3b), and histogram of responses of Overall perceived risk index (S3c) for 10751 participants attending BreastScreen Western Australia between June to December 2023. Participants rated each of the ten body surveillance and body shame questions on a 5-point Likert scale from strongly disagree to strongly agree. The median and interquartile range are shown for each histogram.

# Supplementary Figure S4a: Perceived Risk Latent Variable Model Analyses 1

# Supplementary Figure S4b: Perceived Risk Latent Variable Model Analyses 2

# Supplementary Figure S4c: Body Shame Latent Variable (5 responses) Model Analyses

# Supplementary Figure S4d: Body Surveillance Latent Variable (5 responses) Model Analyses

# Supplementary Figure S4e: Body Shame Latent Variable (3 responses) Model Analyses

# Supplementary Figure S4f: Body Surveillance Latent Variable (3 responses) Model Analyses

**Supplementary Figure S4.** Chi-square residuals with factor scores for modelled response patterns by Graded Response Models (GRMs). Plots represent GRM fits to a) all 3 ordinal perceived risk variables (manifest variables) for all individuals (Supplementary Figure S4a) and separately to data for individuals: b) providing a Numeric score of exactly 50%, with Verbal and Comparative as the manifest variables (Supplementary Figure S4b, GRM 2a); and c) providing a Numeric score other than exactly 50%, with all 3 perceived risk variables as the manifest variables (Supplementary Figure S4b, GRM 2b). Green dashed lines show fitted local regressions demonstrating the trend in residuals, as distributed with model-estimated values of the latent trait. For GRM 2b, the x-axis scale has been reversed to ensure that factor scores are comparable with those estimated for GRM 1 and GRM 2a, in terms of the unobserved overall perceived risk. Similarly, chi-square residuals with factor scores for modelled response patterns by GRMs for an (unobserved) body shame index and body surveillance index using the 5-point Likert scale or following conversion to three responses are shown in Supplementary Figures S4c, S4d, S4e, and S4f.

# Supplementary Material 5_ Spearman’s Rho Correlation Matrix for the study variables

| Variables | 1 | 2 | 3 | 4 | 5 | 6 | 7 | 8 | 9 | 10 | 11 | 12 | 13 |
| --- | --- | --- | --- | --- | --- | --- | --- | --- | --- | --- | --- | --- | --- |
| 1. BMI | 1 (p=NA) |  |  |  |  |  |  |  |  |  |  |  |  |
| 2. Pain Score | -0.04 (p < 0.0001) | 1 (p=NA) |  |  |  |  |  |  |  |  |  |  |  |
| 3. Overall Perceived Risk Index | 0.08 (p < 0.0001) | 0.09 (p < 0.0001) | 1 (p=NA) |  |  |  |  |  |  |  |  |  |  |
| 4. I would be embarrassed for people to know what I really weigh | 0.54 (p < 0.0001) | 0.05 (p < 0.0001) | 0.11 (p < 0.0001) | 1 (p=NA) |  |  |  |  |  |  |  |  |  |
| 5. When I can’t control my weight, I feel like something must be wrong with me | 0.23 (p < 0.0001) | 0.06 (p < 0.0001) | 0.07 (p < 0.0001) | 0.50 (p < 0.0001) | 1 (p=NA) |  |  |  |  |  |  |  |  |
| 6. When I think I am not the size I should be I feel embarrassed | 0.32 (p < 0.0001) | 0.06 (p < 0.0001) | 0.1 (p < 0.0001) | 0.69 (p < 0.0001) | 0.60 (p < 0.0001) | 1 (p=NA) |  |  |  |  |  |  |  |
| 7. I feel embarrassed of myself when I haven’t made an effort to look my best | 0.12 (p < 0.0001) | 0.05 (p < 0.0001) | 0.07 (p < 0.0001) | 0.39 (p < 0.0001) | 0.44 (p < 0.0001) | 0.51 (p < 0.0001) | 1 (p=NA) |  |  |  |  |  |  |
| 8. I rarely think about how I look | 0.01 (p=0.37) | -0.05 (p < 0.0001) | -0.03 (p=0.0017) | -0.18 (p < 0.0001) | -0.22 (p < 0.0001) | -0.28 (p < 0.0001) | -0.28 (p < 0.0001) | 1 (p=NA) |  |  |  |  |  |
| 9. During the day, I think about how I look many times | 0.05 (p < 0.0001) | 0.07 (p < 0.0001) | 0.05 (p < 0.0001) | 0.32 (p < 0.0001) | 0.38 (p < 0.0001) | 0.42 (p < 0.0001) | 0.37 (p < 0.0001) | -0.37 (p < 0.0001) | 1 (p=NA) |  |  |  |  |
| 10. I rarely worry how I look to other people | -0.02 (p=0.031) | -0.06 (p < 0.0001) | -0.05 (p < 0.0001) | -0.25 (p < 0.0001) | -0.28 (p < 0.0001) | -0.36 (p < 0.0001) | -0.34 (p < 0.0001) | 0.41 (p < 0.0001) | -0.41 (p < 0.0001) | 1 (p=NA) |  |  |  |
| 11. I rarely compare how I look with how other people look | -0.03 (p=0.00046) | -0.07 (p < 0.0001) | -0.08 (p < 0.0001) | -0.30 (p < 0.0001) | -0.33 (p < 0.0001) | -0.41 (p < 0.0001) | -0.33 (p < 0.0001) | 0.46 (p < 0.0001) | -0.47 (p < 0.0001) | 0.51 (p < 0.0001) | 1 (p=NA) |  |  |
| 12. I think it is more important that my clothes are comfortable than whether they look good on me | 0.06 (p < 0.0001) | -0.04 (p < 0.0001) | -0.02 (p=0.017) | -0.09 (p < 0.0001) | -0.11 (p < 0.0001) | -0.14 (p < 0.0001) | -0.18 (p < 0.0001) | 0.33 (p < 0.0001) | -0.25 (p < 0.0001) | 0.28 (p < 0.0001) | 0.30 (p < 0.0001) | 1 (p=NA) |  |
| 13. I think more about how my body feels than how my body looks | -0.01 (p=0.28) | -0.06 (p < 0.0001) | -0.04 (p < 0.0001) | -0.24 (p < 0.0001) | -0.23 (p < 0.0001) | -0.30 (p < 0.0001) | -0.25 (p < 0.0001) | 0.38 (p < 0.0001) | -0.37 (p < 0.0001) | 0.36 (p < 0.0001) | 0.43 (p < 0.0001) | 0.43 (p < 0.0001) | 1 (p=NA) |

BMI = Body Mass Index

NA = Not Applicable
